# Soil Characteristic Comparison of Fenced and Grazed Riparian Floodplain Wetlands in the Typical Steppe Region of the Inner Mongolian Plateau, China

**DOI:** 10.1155/2014/765907

**Published:** 2014-05-19

**Authors:** Lixin Wang, Huamin Liu, Yuhong Liu, Jianwei Li, Hongbo Shao, Wei Wang, Cunzhu Liang

**Affiliations:** ^1^College of Environment and Resources, Inner Mongolia University, Hohhot 010021, China; ^2^College of Life Sciences, Inner Mongolia University, Hohhot 010021, China; ^3^Yantai Institute of Coastal Zone Research, Chinese Academy of Sciences, Yantai 264003, China; ^4^BERIS Engineering and Research Corporation, Baotou 014010, China

## Abstract

In recent decades, degradation of ecosystem in the steppe region of the Inner Mongolia Plateau, especially in riparian floodplain wetlands, has become a significant ecological crisis. Not uncommonly, with the increasing of livestock in the Inner Mongolian steppe region, a riparian floodplain wetland is becoming a hotspot area of grazing for local herdsmen. Hence, it is essential to understand degradation mechanisms of riparian floodplain wetland ecosystems caused by extensive grazing. In this study, the spatial distribution of soil compaction, salinity, total nitrogen, total phosphorus, organic carbon, and microbial biomass C and N were investigated. The results showed that grazing led to an increase in soil compaction and soil surface salinity, which significantly lowered levels of total N, P, and TOC in the soil surface. Grazing decreased soil microbial biomass C and N concentration in the lower riparian floodplain wetland, whereas it significantly increased soil microbial biomass C and N concentration in the higher riparian floodplain wetland. Elevation differences in the riparian floodplain wetland increased spatial heterogeneity in the soil and thus resulted in different influence of grazing on wetland soils and ecosystem. Therefore, elevation differences and grazing intensity were the main factors controlling soil characteristics in the riparian floodplain wetland of this region.

## 1. Introduction

The restoration and degradation characteristics of degraded riparian wetlands have become an important subject in wetland ecological studies [1–4]. Presently, most studies focused on decreases in wetland area and species succession, underlying degradation mechanisms affected by climate change [5, 6], land use [5–8], and overgrazing [9–14], and changes in nutrient composition of degraded wetlands [15–18].

With the increasing of livestock in the Inner Mongolian steppe region, grazing intensity also increased, which led to grassland degradation at various degrees [19, 20] and greatly decreases productivity [21, 22], hence existing pastures for grazing were not big enough to supply enough food for the livestock, and local people were transferring their livestock from grasslands to adjacent riparian floodplain wetlands [23, 24]. As a result, vegetation and soil physical and chemical properties of riparian floodplain wetland began to degrade, and riparian floodplain wetland ecosystem health was affected by grazing intensity. Although grazing effects on typical steppe ecosystems have been thoroughly studied, which includes biodiversity, grassland productivity, soil physical and chemical properties, and changes in the succession of degraded plant communities [24–31], few studies were carried on the influences of grazing on adjacent riparian wetland soil and their underlying mechanisms of degradation in this region.

In this study, a fenced conservation and a degraded grazing riparian floodplain wetland with similar initial environmental conditions were chosen as experiment sites in the riparian floodplain wetland of the Xilin River in the typical steppe region in the Inner Mongolian Plateau. Soil properties and its nutrient contents of grazed and fenced river floodplain wetlands were investigated, and these characteristics were compared in order to provide a theoretical foundation for sustainable development and managing of wetland natural resources in the steppe regions of the Inner Mongolian Plateau. We hypothesized that (1) direct grazing and trampling would increase soil compaction and decrease soil carbon content and (2) various elevations would affect levels of inundation in riparian floodplain wetland, and this would change distribution of grazing intensity, whereas vegetation compositions and soil properties in different locations of floodplain wetland also would be altered accordingly.

## 2. Study Areas

**Figure 1:**
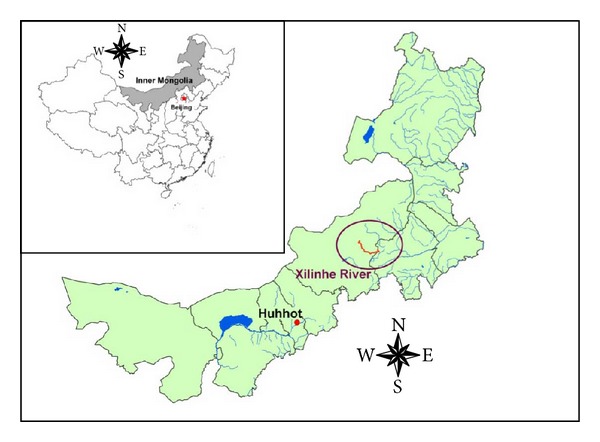
Location of Xilin River in Inner Mongolia, China.

The Xilin River, one of the major inland rivers, flows from southeast to northwest, and its floodplain with a total area of 10,000 km^2^ is located at the eastern edge of the Xilingol High Plain in the middle of the Inner Mongolian Plateau (Figure 1). The elevation of the Xilin River watershed varies from 1,000 m to 1,500 m and decreases from east to west. The climate in the Xilin River watershed is described as a continental temperate steppe climate. The vegetation consists of typical steppe vegetation and the soil is mainly dark Kastanozems and light Kastanozems. The local economy is livestock-based.

**Figure 2:**
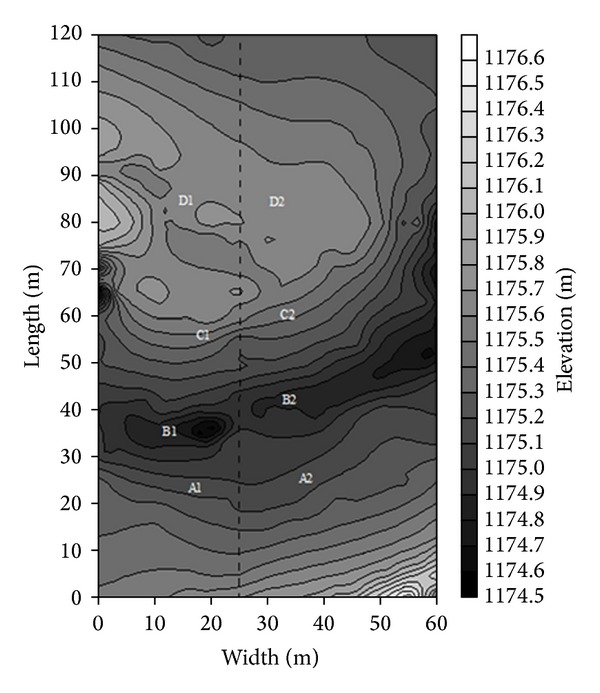
Topographical map of the study sites.

Experiment sites, including grazed site and fenced site, were designed in the floodplain of the middle reach of the Xilin River 500 m away from the west of the Inner Mongolian Grassland Ecosystem Research Station of the Chinese Academy of Sciences with an elevation of 1,177 m (43°37′40′′ N, 116°41′11′′ E). Overgrazing and trampling caused by nearby livestock existed on the degraded grazed site, whereas the fenced site was enclosed to exclude grazing livestock. The two sites were separated by a fence (at 35 m on the *x*-axis in Figure 2) and had similar initial environmental conditions. To eliminate the influence of microtopography on soil physical and chemical properties, wetland soil characteristics at locations A, B, C, and D with similar topography were compared in the two sites (Figure 2). The species compositions of the fenced and grazed wetland were listed in Table 1.

## 3. Methods

### 3.1. Field Sampling and Laboratory Analysis

The samples of wetland soil were collected from 0–10 cm, 10–20 cm, 20–30 cm, 30–40 cm, and 40–50 cm by a soil auger with three replicates at each depth during the growing season in 2010. Soil compaction was measured by using the Soil Hardness Meter 6110 FS. Three soil samples obtained by cutting ring with a volume of 100 cm^3^ were taken to the laboratory and dried at 108°C until constant weight in order to measure soil bulk density. Soil samples from different depths, from which plant tissues were removed, were air-dried and the remaining soil materials were then sieved through a 2 mm filter. About one-fourth of each sample was ground using a globe grinder RetschRM100, passed through a number 100 sieve (*d* = 150 *μ*m), and then stored in glass vials for analyzing its chemical and physical properties. Soil total nitrogen (N) content, soil total phosphorus (P) content, and soil organic carbon (TOC) were measured by using a UDK 142 + DK20 Kjeltec Auto Analyzer, the Molybdenum blue colorimetric method [32], and a Liqui TOC analyzer (Germany) [33], respectively. C and N contents of soil microbial biomass were determined by using the chloroform fumigation-K_2_SO_4_ extraction method [34]. Soil salinity was tested by using a Spectrum 2265 FS [35], and pH was determined using a Spectrum IQ150 [36].

### 3.2. Data Analysis

Data processing of soil compaction, soil nutrient, and soil microbial biomass C and N were performed using Microsoft Excel 2007 and GraphPad Prism 5.0.

One-way ANOVA was used to evaluate the differences among soil salinity of the grazed and fenced floodplain wetlands by SPSS 16.0.

## 4. Results

### 4.1. Comparison of Soil Compaction in Fenced and Grazed Wetlands

**Figure 3:**
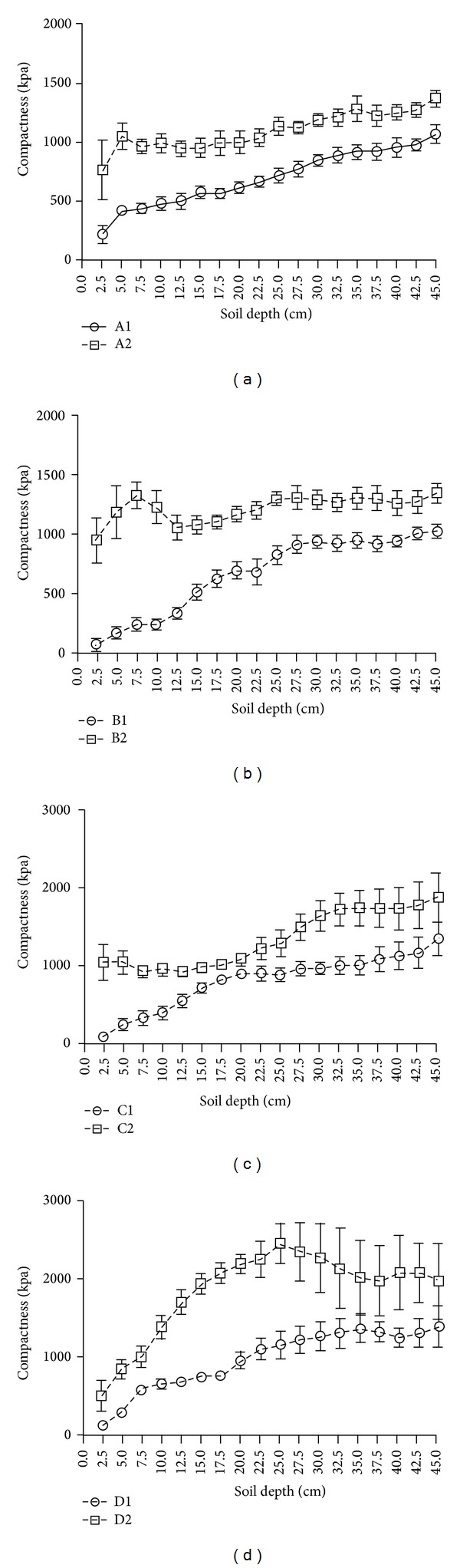
Spatial distribution of soil compaction.

Grazing increased soil compaction (Figure 3), especially in the soil outside the fence. The soil compaction layer found in the A2 association was located at a depth of 5 cm from the soil surface, and the level of compaction increased with depth. The soil compaction layer found in the B2 association existed at a depth of 7.5 cm from the soil surface, and the level of soil compaction did not change much with depth. No soil compaction layer was found in the C2 and D2 association. Soil compaction in C2 association increased with depth. The greatest compaction in D2 was located at a depth of 27.5 cm. There were similar trends that soil compaction increased with depth in A1, B1, C1, and D1 association of fenced wetlands. Generally, the soil compaction in wetlands C and D was more than in wetlands A and B.

### 4.2. Spatial Distribution of Soil Salinity in Fenced and Grazed Wetlands

**Figure 4:**
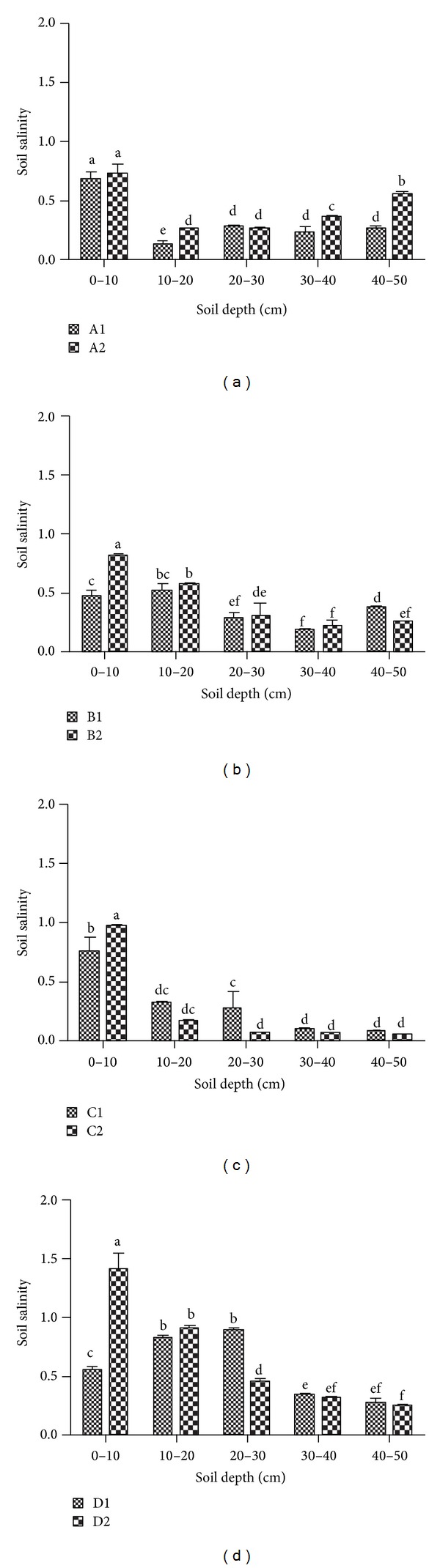
Spatial distribution of soil salinity.

Soil salinity of the top 0–10 cm in the grazed sites was significantly greater than that of the fenced sites except for the same salinity in sites A1 and 2, while there were no significant differences in soil salinity of most other soil layers in grazed and fenced sites (Figure 4). Generally, with soil depth increasing, soil salinity decreased in fenced and grazed sites.

### 4.3. Spatial Distribution of Soil Total N, Soil Total P, and Soil Organic C

**Figure 5:**
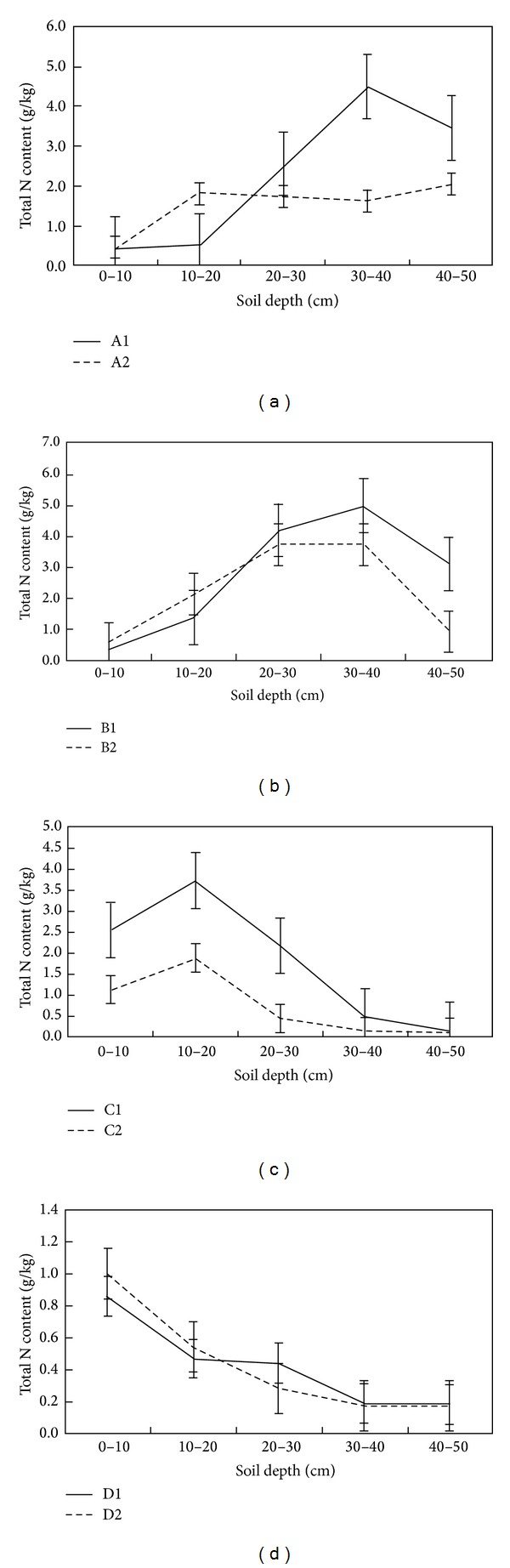
Spatial distribution of soil total N content.

**Figure 6:**
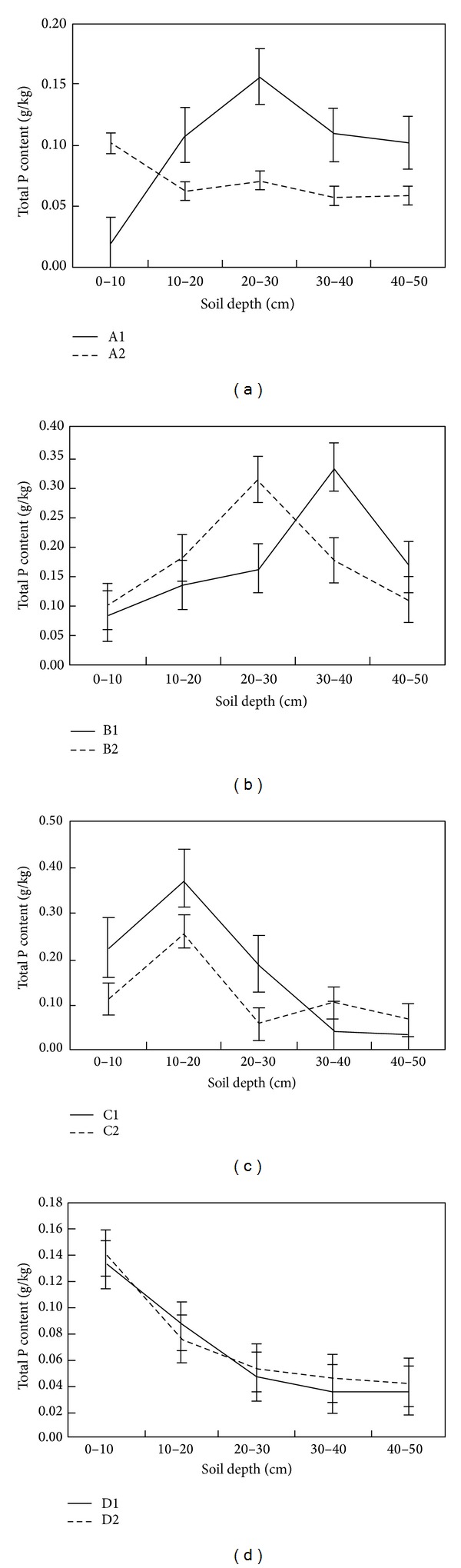
Spatial distribution of soil total P content.

**Figure 7:**
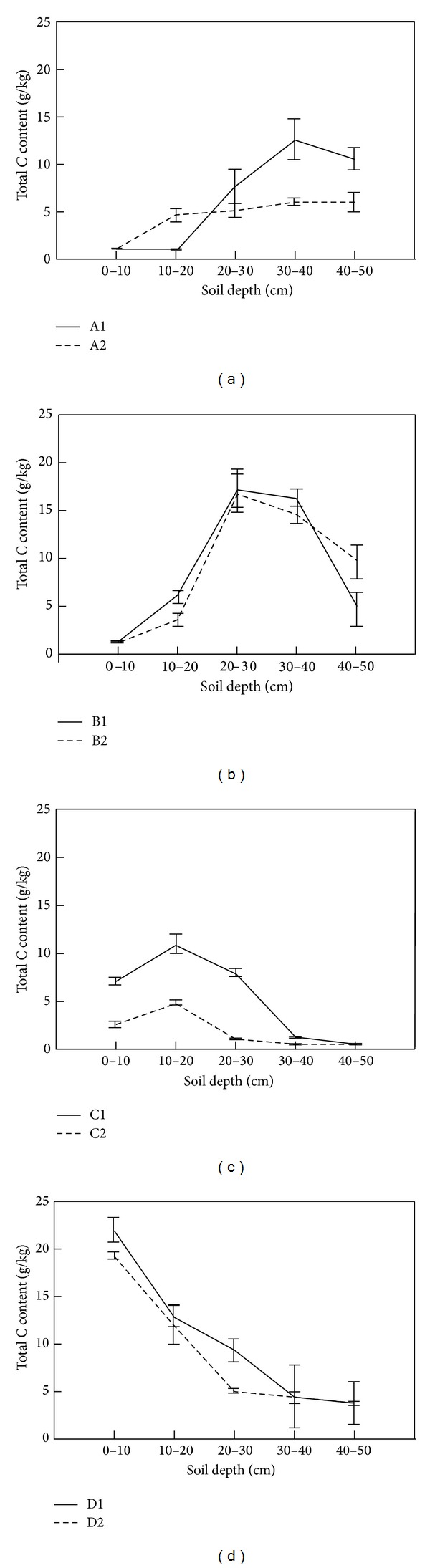
Spatial distribution of soil total C content.

The soil total C, N, and P vertical profiles were similar at the fenced and grazed sites except for in the associations of A1 and A2, hence grazing did not affect the vertical distribution of soil total N and P; that is, Maximum soil total C, N, and P content was found at a depth of 20–40 cm in B1 and B2, at a depth of 10–20 cm in C1 and C2, and at a depth of 0–10 cm in D1 and D2, whereas Maximum soil total C, N, and P content in A1 was different from in A2 (Figures 5, 6, and 7).

### 4.4. Comparison of Soil Microbial Biomass C and N

**Figure 8:**
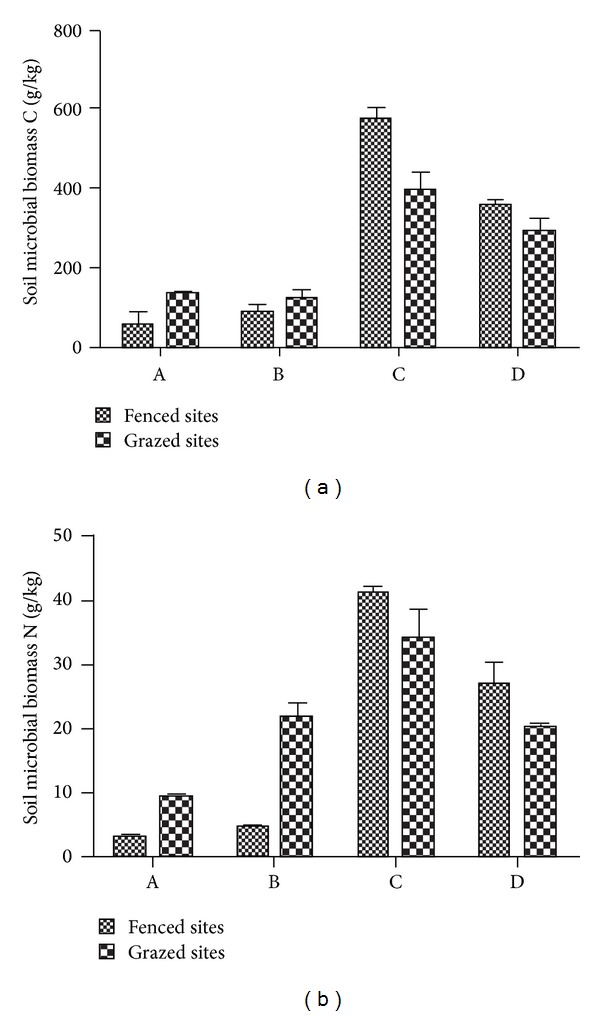
Spatial distribution of soil microbial biomass C and N.

In the low elevation floodplain wetlands A and B, soil microbial biomass C and N content of the grazed site was greater than that of the fenced site. However, in the transition zone and the high elevation floodplain wetlands C and D, soil microbial biomass C and N content of the grazed site was significantly lower than that of the fenced site (Figure 8). Moreover, contents in soil microbial biomass C and N in the high elevation wetlands C and D were more than in the low elevation wetlands A and B.

## 5. Discussion

Some studies have reported that grazing and trampling increased soil compaction and soil salinity (e.g., [37, 38]), changed nutrient conditions (e.g., [39–41]), and resulted in a smaller size of individual wetland plant with lower biomass [42–44]. These phenomena also existed in our study results.

Our study further proved that grazing increased soil compaction in the grazed wetland more significantly than in the fenced wetland. Moreover, a compaction layer was formed in the soil surface of sites A2 and B2, while no soil compaction layers were found in the fenced wetlands. This was because undecomposed remains of plant litters accumulated fast in sites A and B, and trampling of livestock caused these remains to form a compaction layer. The large pores in the wetland soil are the main passage for transporting water, the reduction of which limits the transportation of water and nutrients to the roots [45, 46]. Generally, trampling reduced soil pore space in the wetland and increased soil compaction [38, 47, 48]. In the grazed sites A to D, grazing and trampling reduced soil pore space and lowered soil water content in site D more than in the other sites, which were critical in changing soil physical properties.

Some studies investigated the response of wetland plants to grazing and found that reactions were dependent on hydrological conditions and grazing intensity [9, 49–51]. Soil water content was thus the main limiting factor for plant productivity [21, 52], and Pietola et al. [53] found that trampling at wetter conditions made surface soil looser and increased the air permeability and saturated hydraulic conductivity, while in deeper soil layers it was contrary. In this study, the floodplain wetlands A to D showed distinctive characteristics in the variety of soil compaction with increasing depth under the influence of grazing, and especially in site D, grazing effects were similar to the findings of Pietola et al. [53]. Grazing could decrease litter accumulation and cause plant functional types change, and heavy grazing would produce higher salinity and less biomass [37], which were obvious in our studies. Our studies further showed that grazing promoted the accumulation of soil salinity of the top 0–10 cm, and C, N, and P accumulation increased from shallow soil in sites D and C to deep soil on sites A and B. This was attributed to a decrease in soil water content from sites A and B to site D, which was caused by elevation of different sites. Moreover, compositions of plant species varied from sites A to D, and the abundance of tall and rhizomatous species decreased. The phenomenon of individual plant miniaturization revealed that grazing disturbed plant growth. Hence, depressional wetlands such as sites A and B in riparian floodplain benefited from organic C and nutrient accumulation and increased plant species richness.

The shift in elevation of riparian floodplain wetlands added to the spatial heterogeneity of wetland soil, which was also shown in the variations of soil microbial biomass C and N in this study. Grazing intensity increased soil microbial biomass C and N clearly [54, 55] and stagnant flood conditions also decreased microbial biomass [56], which explained that higher soil microbial biomass C and N existed in the higher elevation wetlands C and D for their higher grazing intensity and fewer inundation. The decrease in the number of soil pores limited the growing space for microbes and decreased microbial respiration [57]. The reduction in microbes broke efficient belowground cycles and thus lowered belowground productivity, that is, less root biomass [58, 59]. This phenomenon was obvious in sites C and D. If extensive grazing still continued, plant productivity of the riparian wetland would further decrease and eventually result in extreme ecosystem degradation [19, 29]. Wetland degradation caused less water infiltration, greater surface runoff, and more soil nutrient loss [60–63]. Therefore, grazing also lowered soil total N and total P content in the wetland [37, 64].

All of these changes of the wetland soil resulting from grazing were consistent with the responses of typical degraded steppe to grazing, including lower soil nutrient contents, more compact soil with a compaction layer at the soil surface, and salinized soil [65–69]. Therefore, we should realize the importance of conserving wetland and grassland ecosystems in Xilin River riparian floodplain wetland and strengthen the management of grazing to restore ecosystem health of riparian floodplain wetland.

## 6. Conclusion

Comparison of the soil in the fenced and grazed riparian floodplain wetland in the steppe region of the Inner Mongolian Plateau showed that grazing and trampling from livestock resulted in a more compact soil with a surface compaction layer and less TOC, total N, and total P concentration. Overgrazing produced more exposed soil surfaces, greater soil surface salinity with a tendency towards salinization, and degradation of wetland vegetation. However, variable elevations of riparian floodplain wetlands formed greater spatial heterogeneity in wetland soil and vegetation composition, and thus different topographical characteristics displayed in the influence of grazing on riparian floodplain wetland. Additionally, wetland plant communities at a higher elevation location in riparian floodplain wetland endured more grazing pressures.

## Figures and Tables

**Table 1:** The composition of plant community in the study sites.

Topography	Fenced			Grazed		
	Plant community	Plant height (cm)	Aboveground biomass (g/m^2^)	Plant community	Plant height (cm)	Aboveground biomass (g/m^2^)
Low floodplain wetlands	*Glyceria spiculosa* + *Poa subfastigiata *(A1)	40.66 ± 12.99	415.31 ± 128.84	*Carex appendiculata* + *Geranium vlassowianum *(A2)	18.63 ± 8.07	329.44 ± 40.17
	*Carex. appendiculata* + *Eleocharis valleculosa *(B1)	47.57 ± 17.07	889.49 ± 57.96	*Leymus chinensis* + *Melilotus officinali*s (B2)	38.67 ± 21.76	483.09 ± 181.44

Transition zone	*Carex*. *appendiculata* + *Glyceria spiculosa *(C1)	34.26 ± 10.46	467.92 ± 144.35	*Glyceria spiculosa + Agrostis gigantea *(C2)	14.03 ± 7.49	262.56 ± 94.45

High floodplain wetlands	*Leymus chinensis + Carex korshinskii *(D1)	26.07 ± 21.49	251.41 ± 126.04	*Leymus chinensis + Artemisia tanacetifolia* (D2)	15.43 ± 9.72	138.77 ± 24.23
